# Integrating multi-omics summary data using a Mendelian randomization framework

**DOI:** 10.1093/bib/bbac376

**Published:** 2022-09-12

**Authors:** Chong Jin, Brian Lee, Li Shen, Qi Long, for the Alzheimer’s Disease Neuroimaging Initiative, for the Alzheimer’s Disease Metabolomics Consortium

**Affiliations:** Department of Biostatistics, Epidemiology and Informatics, Perelman School of Medicine, University of Pennsylvania, Philadelphia, PA 19104, USA; Department of Biostatistics, Epidemiology and Informatics, Perelman School of Medicine, University of Pennsylvania, Philadelphia, PA 19104, USA; Department of Biostatistics, Epidemiology and Informatics, Perelman School of Medicine, University of Pennsylvania, Philadelphia, PA 19104, USA; Department of Biostatistics, Epidemiology and Informatics, Perelman School of Medicine, University of Pennsylvania, Philadelphia, PA 19104, USA

**Keywords:** Mendelian randomization, *P*-value combination, multi-omics data, GWAS, QTL

## Abstract

Mendelian randomization is a versatile tool to identify the possible causal relationship between an omics biomarker and disease outcome using genetic variants as instrumental variables. A key theme is the prioritization of genes whose omics readouts can be used as predictors of the disease outcome through analyzing GWAS and QTL summary data. However, there is a dearth of study of the best practice in probing the effects of multiple -omics biomarkers annotated to the same gene of interest. To bridge this gap, we propose powerful combination tests that integrate multiple correlated $P$-values without assuming the dependence structure between the exposures. Our extensive simulation experiments demonstrate the superiority of our proposed approach compared with existing methods that are adapted to the setting of our interest. The top hits of the analyses of multi-omics Alzheimer’s disease datasets include genes *ABCA7* and *ATP1B1*.

## Introduction

**Figure 1:**
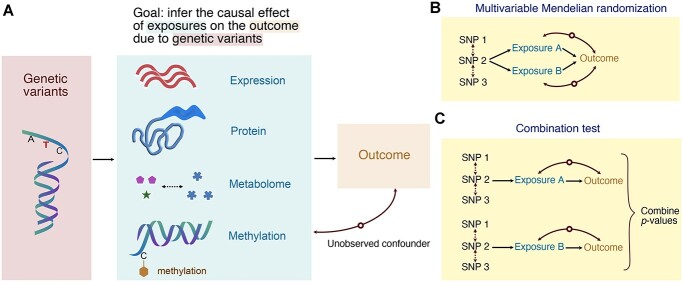
(** A**) A schematic diagram illustrating MR analyses adapted to a multi-omics setting through analyzing QTLs and GWAS summary data to infer the causal effect (or pleiotropic effect) between the exposures and the outcome. (** B**) Multivariable MR is a strategy to interrogate the effect of multiple exposures on the outcome. (** C**) We propose combination tests that combine $P$-values from MR experiments based on multiple exposures.

The importance of Mendelian randomization (MR) has been widely recognized [1], particularly in observational studies where investigators probe the effect of an exposure, i.e. a risk factor, on an outcome using heredity as a natural experiment. There is a large body of literature on MR methods particularly for settings with only one molecular exposure [2–5]. However, there has been very limited work and lacks a systematic investigation and consensus on the optimal MR approach(s) when the exposures consist of multiple omics biomarkers such as transcriptomics, proteomics and metabolomics data (Figure 1A). In this work, we seek to evaluate different strategies, some of which are not proposed before under the setting, to integrate $P$-values when inferring the causal effect (or pleiotropic association) of multiple omics biomarkers on the outcome using an MR framework.

A valid MR investigation rests upon a series of assumptions [6]. These assumptions are as follows.

(i) The genetic variant is associated with the biomarker.
(ii) The genetic variant cannot be associated with any confounder that lies within the biomarker-outcome relationship.
(iii) The genetic variant cannot be associated with the outcome through any pathway other than through the biomarker in question.

The first assumption is fairly easy to satisfy in MR studies. When we employ genetic variants as instrumental variables (IVs) to probe whether a biomarker has a causal effect on an outcome such as disease status [6], we choose genetic variants highly associated with the risk factor so they quality as strong IVs. In contrast, the second and third assumptions are harder to verify due to special challenges in MR: the SNPs are often in linkage disequilibrium (LD) resulting in nebulous interpretations of the association(s) and a large number of candidate exposures may lead to widespread pleiotropy. Thus, our findings of MR analysis need to be interpreted as ‘pleiotropic association(s)’ [7].

To provide a panoramic view of how exposures jointly affect the outcome, instead of having one exposure, we aggregate multiple exposures (Figure 1A). Common examples include different omics biomarkers such as transcriptomics, proteomics and metabolomics [8], multiple tissues [9] and the expression of multiple genes in physical proximity [10]. In this article, we mainly explore aggregating the effects of multiple omics biomarkers, though the model can be readily extended to other situations.

Inspired by how multiple traits in multivariate genome-wide association study (GWAS) are modeled [11], we lay out three strategies for modeling multiple exposures in MR: transformation-based, regression-based and combination tests.

(i) Transformation-based tests require a transformation of the measurement of exposures into a single new synthetic exposure through principal component analysis (PCA) or other dimensionality reduction techniques [12, 13]. Note that as opposed to the other two summary data-based approaches, individual-level data access is necessary to enable the transformation.
(ii) Regression models constitute the backbones of MR in practice. If we ignore the causal relationships between the omics biomarkers, then multivariable MR is the appropriate model since the SNPs have direct effects on the biomarkers [14] (Figure 1B).
(iii) Combination tests circumvent the necessity of modeling the relationship between different exposures, which makes the investigation easier under the perspective of meta-analysis of summary data where the relationship between different exposures is largely unknown (Figure 1C).

We focus the attention on combination tests, which have not been applied to the MR setting. The merit of our novel framework is that it can be applied when the structure between multiple exposures is unknown ahead of time or there is no prior knowledge about the structure in a multi-omics study.

In what follows, we first present an overview of the methods and then assess and compare these methods through simulation experiments and analyses of multi-omics datasets of the study of Alzheimer’s disease.

## Methods

### Existing methods

We begin by describing several existing methods as building blocks toward conducting MR analyses based on multi-omics summary data.

#### Inverse variance-weighted average method

Inverse variance-weighted average method (IVW) is one of the most popular summary-data-based MR methods. While individual-level genetic data are usually considered sensitive and protected, GWAS and quantitative trait loci (QTL) summary statistics are easier to obtain. These summary statistics can then be used to reach a larger sample size and a higher power in a meta-analysis. Here, we assume that the GWAS and QTL summary statistics are generated from different individuals (two-sample setting) and we have effect size estimates, standard error estimates, statistics, and $P$-values for SNPs $j = 1 \ldots p$ as IVs. In our setting, the $p$ SNPs are at most 1M base pairs apart from the gene body so that they count as cis-QTLs of the gene.

When the genetic variants are uncorrelated, the IVW estimate of the causal effect size of the outcome on the exposure $k$ is [6]

$$\begin{align*} & \hat{\theta}_{\mathrm{IVW},k} = \frac{\sum_{j=1}^p \widehat{\beta}_{Xkj} \widehat{\beta}_{Yj} \widehat{\sigma}_{Yj}^{-2}}{\sum_{j=1}^p \widehat{\beta}_{Yj}^2 \widehat{\sigma}_{Yj}^{-2}}, \end{align*}$$

where the standard error of the estimate is

$$\begin{align*} & \mathrm{se}(\hat{\theta}_{\mathrm{IVW},k}) = \sqrt{\frac{1}{\sum_{j=1}^p \widehat{\beta}_{Yj}^2 \widehat{\sigma}_{Yj}^{-2}}}. \end{align*}$$

Here, $\widehat{\beta }_{Xkj}$ is the regression coefficient of the exposure $k$ on the SNP $j$ in the form of QTL and $\widehat{\beta }_{Yj}$ is the regression coefficient of the outcome on the SNP $j$ in the form of GWAS. $\widehat{\sigma }_{Yj}^{2}$ is the standard error of the regression coefficient of the outcome on the SNP $j$.

The omics biomarker $k$ can stand for transcriptomics, proteomics, metabolomics or other omics readouts. This allows us to combine such $P$-values using a method we choose (i.e. Fisher’s combination test, Cauchy combination test, etc.).

#### Generalized least squares method

The generalized least squares method (GLS) builds upon IVW and accounts for remaining LD within SNPs after LD clumping [5]. The notations are the same as they appear in IVW

$$\begin{align*} \widehat{\theta}_{\mathrm{GLS},k} & = (\boldsymbol{\widehat{\beta}}_{Xk}^T \Omega^{-1}\boldsymbol{\widehat{\beta}}_{Xk})^{-1} (\boldsymbol{\widehat{\beta}}_{Xk}^T \Omega^{-1}\boldsymbol{\widehat{\beta}}_{Y}), \\ \mathrm{var}\left(\widehat{\theta}_{\mathrm{GLS},k}\right) & = (\boldsymbol{\widehat{\beta}}_{Xk}^T \Omega^{-1}\boldsymbol{\widehat{\beta}}_{Xk})^{-1}, \end{align*}$$

where the covariance matrix $\Omega $ is constructed from the unsquared LD matrix $R$

$$\begin{align*} &\Omega = (\widehat{\sigma}_{Yi} \widehat{\sigma}_{Yj} r_{ij}) = \begin{bmatrix} \widehat{\sigma}_{Y1} & & \\ &\ddots& \\ & &\widehat{\sigma}_{Yp} \end{bmatrix} R \begin{bmatrix} \widehat{\sigma}_{Y1} & & \\ &\ddots& \\ & &\widehat{\sigma}_{Yp} \end{bmatrix} ,\end{align*}$$

and $r_{ij}$’s are the entries in the unsquared LD matrix $R$.

#### Summary data-based MR

The notable method Summary data-based MR (SMR) was introduced in [7]. The major distinction compared with IVW is that it uses a second-order weight and that information from only one SNP is needed.

Under the assumption that the GWAS $z$-value $z_{Y}$ and QTL $z$-value $z_{Xk}$ are estimated using independent samples, we have an approximate $\chi ^2$ test statistic with one degree of freedom

$$\begin{align*} &T_{\mathrm{SMR,}k} = \frac{z_{Y}^2 z_{Xk}^2}{z_{Y}^2 + z_{Xk}^2}. \end{align*}$$

To extend SMR statistics to multiple exposures, we separate the calculation of SMR statistics (one SNP and one omic biomarker) [7] and then combines the $P$-values. In the method labeled as ‘allSNPs’, $P$-values of SMR from all combinations of SNPs and omics biomarkers are aggregated, while in the method labeled as ‘singleSNP’, for each omic biomarker, the SNP with the strongest QTL is chosen to derive a $P$-value of SMR. These $P$-values from different biomarkers are aggregated.

#### Generalized summary data-based MR

The generalized summary data-based MR (GSMR) method was introduced in [15] to extend SMR by introducing multiple genetic variants that can be correlated to increase the power.

Let

$$\begin{align*} &\boldsymbol{\widehat{\theta}}_k = (\widehat{\theta}_{k1}, \widehat{\theta}_{k2}, \ldots, \widehat{\theta}_{km})^T = \left(\frac{\widehat{\beta}_{Y1}}{\widehat{\beta}_{Xk1}}, \frac{\widehat{\beta}_{Y2}}{\widehat{\beta}_{Xk2}}, \ldots, \frac{\widehat{\beta}_{Yp}}{\widehat{\beta}_{Xkp}} \right)^T,\end{align*}$$

$$\begin{align*} \mathrm{var}\left(\widehat{\theta}_{kj}\right) = \frac{\widehat{\beta}_{Yj}^2}{\widehat{\beta}_{Xkj}^2} \left[ \frac{\mathrm{var}(\widehat{\beta}_{Xkj})}{\widehat{\beta}_{Xkj}^2} + \frac{\mathrm{var}(\widehat{\beta}_{Yj})}{\widehat{\beta}_{Yj}^2} - \frac{\mathrm{var}(\widehat{\beta}_{Xkj})^2}{\widehat{\beta}_{Xkj}^4} \right], \end{align*}$$

$$\begin{align*} \mathrm{cov}\left(\widehat{\theta}_{ki}, \widehat{\theta}_{kj} \right) & = \frac{r_{ij}\sqrt{\mathrm{var}(\widehat{\beta}_{Yi})\mathrm{var}(\widehat{\beta}_{Yj})}}{\widehat{\beta}_{Xki}\widehat{\beta}_{Xkj}} + \frac{\widehat{\beta}_{Yi}\widehat{\beta}_{Yj}}{\widehat{\beta}_{Xki}\widehat{\beta}_{Xkj}} \\ & \left[\frac{r_{ij}\sqrt{\mathrm{var}(\widehat{\beta}_{Xki})\mathrm{var}(\widehat{\beta}_{Xkj})}}{\widehat{\beta}_{Xki}\widehat{\beta}_{Xkj}} - \frac{\mathrm{var}(\widehat{\beta}_{Xki})\mathrm{var}(\widehat{\beta}_{Xkj})}{\widehat{\beta}_{Xki}^2 \widehat{\beta}_{Xkj}^2} \right],\\ V_k & = \left(\mathrm{cov}\left(\widehat{\theta}_{ki}, \widehat{\theta}_{kj} \right)\right)_{p\times p}. \end{align*}$$

$$\begin{align*} &\widehat{\theta}_{\mathrm{GSMR},k} = (\mathbf{1}^T V_k^{-1} \mathbf{1} )^{-1} \mathbf{1}^T V_k^{-1} \boldsymbol{\widehat{\theta}_k}, \end{align*}$$

$$\begin{align*} &\mathrm{var}\left(\widehat{\theta}_{\mathrm{GSMR},k}\right) = (\mathbf{1}^T V_k^{-1} \mathbf{1} )^{-1}, \end{align*}$$

$$\begin{align*} &T_{\mathrm{GSMR},k} = \widehat{\theta}_{\mathrm{GSMR},k}^2 / \mathrm{var}\left(\widehat{\theta}_{\mathrm{GSMR},k}\right). \end{align*}$$

Here, $\widehat{\beta }_{Xkj}$ is the regression coefficient of the exposure $k$ on the SNP $j$ in the form of QTL and $\widehat{\beta }_{Yj}$ is the regression coefficient of the outcome on the SNP $j$ in the form of GWAS. $V_k$ is the covariance matrix for the effect size estimates derived from multiple genetic variants.

The test statistic, $T_{\mathrm{GSMR},k}$, follows a $\chi ^2$ distribution with 1 degree of freedom under the null. Note that GSMR needs multiple strong—and weakly correlated—IVs to work. As is implemented in the GSMR R package, the default filtering conditions on the SNPs are $r^2 < 0.95$ and $\chi ^2> 10$ for the SNPs, and after the filtering, at least 10 SNPs are recommended. Otherwise, the method often cannot work due to the covariance matrix being not invertible.

#### Multivariable MR

When a genetic variant is causally linked to multiple related exposures, multivariable MR may be adopted as the primary analysis strategy [2]. Multivariable MR under one sample and two sample settings is extensively discussed in [16]. In multivariable MR, the effect sizes $\theta _k$’s are coefficients in the regression model

$$\begin{align*} &\widehat{\beta}_{Yj} = \sum_{k=1}^m \theta_k \widehat{\beta}_{Xkj} + \epsilon, \quad \mathrm{weights} = \widehat{\sigma}_{Yj}^{-2}, \end{align*}$$

where $\widehat{\beta }_{Yj}$ is the regression coefficient of the outcome on SNP $j$ and $\widehat{\beta }_{Xkj}$ is the regression coefficient of exposure $k$ the on SNP $j \in \{1 \ldots p\}$.

To perform a joint test whether there is any exposure $k$ having a pleiotropic effect on the outcome using multivariable MR, we test the null hypothesis of

$$\begin{align*} &\theta_1 = \theta_2 = \cdots = \theta_k = \cdots = \theta_m = 0. \end{align*}$$

This results in a test with $m$ degrees of freedom.

Multivariable MR can be viewed as an extension of the IVW method (and the GLS method in presence of LD) [17]. More IVs (variants) are needed than exposures. Otherwise, the fitted values of different exposures by plugging in the IVs will not constitute a full rank matrix. While multivariable MR generally requires uncorrelated variants, it can be extended to incorporate correlated variants using GLS [5, 10].

The interpretation of the effect size estimate differs from the interpretation when we only have one exposure. Multivariable MR quantifies the direct effect of an exposure on the outcome as opposed to the total effect of an exposure on the outcome using conventional univariable MR [16].

### Proposed method: combining omics biomarkers annotated to one gene

**Figure 2:**
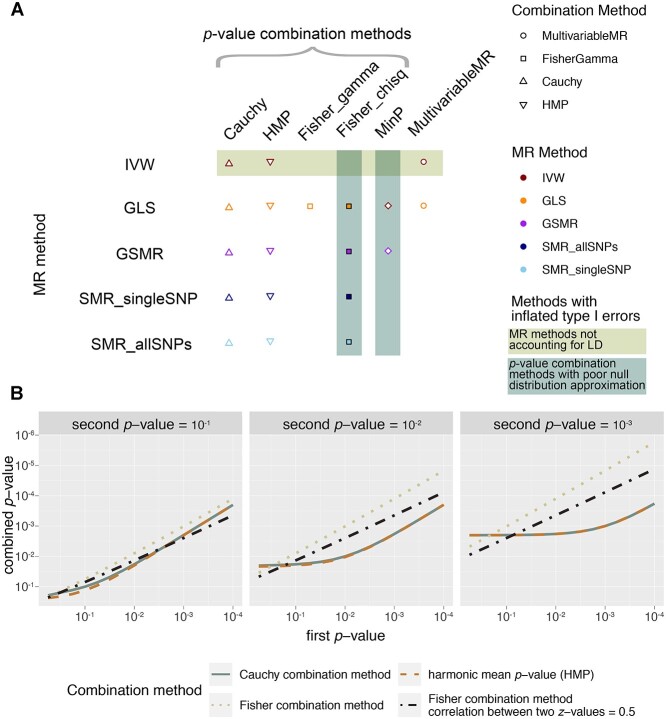
(** A**) Overview of MR methods and combination methods illustrated in our benchmark. Note that Fisher_gamma is only implemented for GLS, and MultivariableMR can be combined with either IVW and GLS. More results of Fisher_chisq and MinP-related methods can be found in the online supporting data repository. (** B**) $P$-value combination methods when combining two $P$-values. The first $P$-value is plotted on the $x$-asis and the second $P$-value is plotted along the panels. The function in the Cauchy combination method (grey solid line) is very similar to the function in harmonic mean $P$-value (brown dashed line) albeit the latter one is a bit more conservative; both methods are valid regardless of the correlation between $P$-values. The Fisher combination method using chi-squared approximation ‘Fisher_chisq’ (beige dotted line), more liberal than others, was originally proposed for independent tests and can be adapted to ‘Fisher_gamma’ where the covariance structure of the $z$-values underlying the $P$-values is known (black dot-dashed line; correlation between two $z$-values is 0.5 as an example).

We propose combination tests, which have not been applied to the multi-omics MR setting before, to investigate the causal effects of multiple omics biomarkers on an outcome. When conducting the combination tests, we first test the effect of each omics biomarker on the outcome separately using an MR method we covered above and then combine the $P$-values using the methods detailed below. The combinations of strategies are listed in Figure 2A.

- Cauchy combination test [18–20]: The method calculates a $P$-value for each omic biomarker and then combines the $P$-values using Cauchy combination function and Cauchy distribution approximation
  $$\begin{align*} &T_{\mathrm{Cauchy}} = \sum_{k=1}^m w_k \tan \{(0.5 - p_k)\pi\}, \end{align*}$$
  where $w_k$’s are weights determined externally. A natural choice of equal weights $w_k = 1/m$ is used here.
  Regardless of the correlation structure between $P$-values $p_k$, $T_{\mathrm{Cauchy}}$ can be approximated using Cauchy distribution, and the $P$-value of the Cauchy combination test is
  $$\begin{align*} &0.5 - \arctan \left(T_{\mathrm{Cauchy}} / \sum_{k=1}^m w_k\right) / \pi. \end{align*}$$
  Note that when $T_{\mathrm{Cauchy}}$ is very small, we may use other approximations during computation for numerical issues.
- Harmonic mean $P$-value (HMP): HMP is a $P$-value combination method originated from Bayesian model averaging offered as an alternative to Fisher’s method when the $P$-values are correlated [21]
  $$\begin{align*} &T_{\mathrm{HMP}} = \frac{\sum_{k=1}^m w_k}{\sum_{k=1}^m w_k / p_k}. \end{align*}$$
  The HMP test statistics are then compared with a Stable distribution with tail index $\lambda = \beta = 1$ to derive the $P$-value.
- Minimum $P$-value (MinP): The statistic is
  $$\begin{align*} &T_{\mathrm{MinP}} = \min_{k \in \{1 \ldots m\}} p_k, \end{align*}$$
  and when only summary data are available, we ignore the correlation between $P$-values and take the statistic $T_{\mathrm{MinP}}$ as the $P$-value.
- Fisher_chisq: This method calculates a $P$-value for each omic biomarker and then combines the $P$-values using Fisher combination function and the chi-squared approximation
  $$\begin{align*} &T_{\mathrm{Fisher}} = - 2 \sum_{k = 1}^m \ln (p_k). \end{align*}$$
  The Fisher combination statistics are compared with a chi-squared distribution of $2m$ degrees of freedom when the $P$-values are independent.
  When the $P$-values are not independent and we know the association between the $P$-values (can be further approximated using the correlation between the measurements of omics biomarkers), a gamma distribution approximation would be more appropriate, which is discussed below [11, 22, 23].
- Fisher_gamma: We propose the method that combines $P$-values from the GLS method for each omic biomarker. The combination of $P$-values involves the Fisher combination function and gamma distribution approximation of the null hypothesis given the correlation between the summary $z$-statistics [11].
  When we need to combine $P$-values from multiple omics biomarkers using GLS to estimate the effect of pleiotropic association, we first let
  $$\begin{align*} & \boldsymbol{\widehat{\gamma}}_{Xk} = \left(\frac{\widehat{\beta}_{Xk1}}{\widehat{\sigma}_{Y1}}, \ldots, \frac{\widehat{\beta}_{Xkp}}{\widehat{\sigma}_{Yp}}\right)^T, \end{align*}$$
  $$\begin{align*} &\boldsymbol{z}_{Y} = \left(\frac{\widehat{\beta}_{Y1}}{\widehat{\sigma}_{Y1}}, \ldots, \frac{\widehat{\beta}_{Yp}}{\widehat{\sigma}_{Yp}}\right)^T. \end{align*}$$
  The covariance matrix of the GWAS $z$-values $\boldsymbol{z}_Y$ can be approximated using the LD matrix $R$ [11]
  $$\begin{align*} & \mathrm{cov}\left(\boldsymbol{z}_Y\right) = R. \end{align*}$$
  When the SNPs can be deemed as adequately strong IVs, we employ first-order variance estimates where $\widehat{\sigma }_{Xkj}$ approaches zero and $\gamma _{Xk_j}$ can be approximated by a constant vector [24–27].
  Plugging in $\boldsymbol{\gamma }_{Xk}$ and $\boldsymbol{z}_{Y}$ to the GLS estimator, we have
  $$\begin{align*} \widehat{\theta}_{\mathrm{GLS},k} & = (\boldsymbol{\widehat{\gamma}}_{Xk}^T R^{-1}\boldsymbol{\widehat{\gamma}}_{Xk})^{-1} (\boldsymbol{\widehat{\gamma}}_{Xk}^T R^{-1}\boldsymbol{z}_{Y}), \\ \mathrm{var}\left(\widehat{\theta}_{\mathrm{GLS},k}\right) & = (\boldsymbol{\widehat{\gamma}}_{Xk}^T R^{-1}\boldsymbol{\widehat{\gamma}}_{Xk})^{-1}, \\ z_{\mathrm{GLS},k} & = \frac{\widehat{\theta}_{\mathrm{GLS},k}}{\sqrt{\mathrm{var}\left(\widehat{\theta}_{\mathrm{GLS},k}\right)}}, \end{align*}$$
  and
  $$\begin{align*} & \mathrm{cov} \left( z_{\mathrm{GLS,}k_1}, z_{\mathrm{GLS},k_2} \right) \\ = & \mathrm{cov} \left(\frac{\boldsymbol{\widehat{\beta}}_{X{k_1}}^T R^{-1}\boldsymbol{z}_{Y}}{\sqrt{\boldsymbol{\widehat{\beta}}_{X{k_1}}^T R^{-1}\boldsymbol{\widehat{\beta}}_{X{k_1}}}}, \frac{\boldsymbol{\widehat{\beta}}_{X{k_2}}^T R^{-1}\boldsymbol{z}_{Y}}{\sqrt{\boldsymbol{\widehat{\beta}}_{X{k_2}}^T R^{-1}\boldsymbol{\widehat{\beta}}_{X{k_2}}}} \right)\\ = & \left(\frac{\boldsymbol{\widehat{\beta}}_{X{k_1}}^T R^{-1}}{\sqrt{\boldsymbol{\widehat{\beta}}_{X{k_1}}^T R^{-1}\boldsymbol{\widehat{\beta}}_{X{k_1}}}} \right) R \left(\frac{\boldsymbol{\widehat{\beta}}_{X{k_2}}^T R^{-1}}{\sqrt{\boldsymbol{\widehat{\beta}}_{X{k_2}}^T R^{-1}\boldsymbol{\widehat{\beta}}_{X{k_2}}}} \right)^T. \end{align*}$$
  Only a submatrix of $R$ referring to clumped SNPs under omics biomarkers $k_1$ and $k_2$ are needed in the computation of the covariance matrix instead of the LD estimates across all SNPs.After we have an approximation of the covariance matrix between the estimates for different omics biomarkers, we apply the gamma distribution approximation under the null hypothesis given the correlation between the summary $z$-statistics as described in Yang *et al.* [11]. This concludes our proposed method of using Fisher combination function and gamma distribution approximation of the null hypothesis to combine $P$-values representing multiple omics biomarkers obtained from GLS.

Cauchy combination method and HMP, both being valid regardless of correlation between $P$-values, give similar combined $P$-values with Cauchy combination method having a slight edge. While the small $P$-value-producing Fisher combination method is invalid when there is correlation between the two $z$-values as results from Mendelian randomization, Fisher combination methods with a known correlation can strike a trade-off between retaining a small combined $P$-value and relaxing the correlation structure assumptions (Figure 2B).

## Simulation

### Simulation setup

To assess different MR frameworks when combining summary data from multiple omics biomarkers, we perform a comprehensive study of them under different simulation setups. In each simulation setup, we first simulate individual-level SNPs, multi-omics data and outcome labels. Then, the summary statistics are computed by fitting regression models and are fed to the computational methods that provide a $P$-value of whether there is any pleiotropic association between the omic biomarker (exposure) and the outcome. The procedure is repeated 10 000 times and the empirical power and type I error are recorded for each method in each simulation setup.

The total number of subjects in the simulation was chosen to be 500 to represent a typical sample size in a multi-omics study (i.e. the ADNI data used in the real data analysis example). While straightforward to simulate 500 subjects when the outcome is continuous, it requires down-sampling to get the desired number of case and control subjects when the outcome is binary. To generate individual-level data of 250 case and 250 control subjects with a targeted level of subject overlap between QTL and GWAS datasets, we first simulate a population with a much larger number of samples (up to 5000) and generate the outcome label $Y$. We pick 250 case and 250 control subjects from the simulated population to use as samples to generate the GWAS summary statistics. We also pick 250 control subjects with a pre-specified level of overlap (0, 0.5, 1) with the aforementioned 250 control subjects from the simulated population to use as samples to generate the QTL summary statistics.

We assume the SNP effects on omics biomarkers $X_1$, $X_2$ and $X_3$ are constant. We have additional simulations where the SNP effects on omics biomarkers follow an i.i.d. beta distribution detailed in Supporting Information Web Appendix B.

We consider two settings: horizontal pleiotropy where the exposures are in parallel and vertical pleiotropy where the exposures are in series (Figure 5). Under vertical pleiotropy, let $Y_C$ be the continuous outcome and $Y_B$ be the binary outcome. For each individual, the data are generated as below

$$\begin{align*} Z_{j} & \sim \mathrm{Binom}(2, 0.3) \ \mathrm{for} \ j = 1,\ldots,p,\\ r^2_{ij} & = 0, 0.01, \mathrm{or} \ 0.2 \ \mathrm{for} \ i \neq j,\\ X_{1} & = \frac{\alpha}{p} \sum_{j=1}^{p} Z_{j} + U + \epsilon_{X_{1}}, \\ X_{2} & = 2 X_{1} - U + \epsilon_{X_{2}}, \\ X_{3} & = - 0.5 X_{2} + U + \epsilon_{X_{3}}, \\ Y_B & \sim \mathrm{Binom}(1, \tau),\ \mathrm{where} \ \mathrm{logit}(\tau) = -2 + \beta_X X_{3} - U,\\ Y_C & = \beta_X X_{3} - U + \epsilon_{Y},\\ U & \sim \mathcal{N} (0, 1), \ \epsilon_{X_{k}} \sim \mathcal{N} (0, 1), \ \epsilon_{Y} \sim \mathcal{N} (0, 1) \ \mathrm{independently}. \end{align*}$$

Under horizontal pleiotropy, let $Y_C$ be the continuous outcome and $Y_B$ be the binary outcome. For each individual, the data are generated as below

$$\begin{align*} Z_{j} & \sim \mathrm{Binom}(2,\ 0.3) \ \mathrm{for} \ j = 1,\ldots,p,\\ r^2_{ij} & = 0, 0.01, \mathrm{or} \ 0.2 \ \mathrm{for} \ i \neq j,\\ X_{1} & = \frac{\alpha}{p} \sum_{j=1}^{p} Z_{j} + U + \epsilon_{X_{1}}, \\ X_{2} & = -\frac{\alpha}{p} \sum_{j=1}^{\left\lfloor p/2 \right\rfloor} Z_{j} - U + \epsilon_{X_{2}}, \\ X_{3} & = \frac{\alpha}{p} \sum_{j=\left\lfloor p/2 \right\rfloor + 1}^{p} Z_{j} - U + \epsilon_{X_{3}}, \\ Y_B & \sim \mathrm{Binom}(1, \tau),\ \mathrm{where} \ \mathrm{logit}(\tau) = -2 + \beta_X \sum_{k=1}^3 X_{k} + U,\\ Y_C & = \beta_X \sum_{k=1}^3 X_{k} + u_i + \epsilon_{Y},\\ U & \sim \mathcal{N} (0, 1), \ \epsilon_{X_{k}} \sim \mathcal{N} (0, 1), \ \epsilon_{Y} \sim \mathcal{N} (0, 1) \ \mathrm{independently}. \end{align*}$$

Then, we generate the summary data, which are the input of the MR analyses. For the association between exposure $X_k$ and genetic variant $Z_j$, we fit the regression model to get effect size estimate $\widehat{\beta }_{Xkj}$ and standard error estimate $\widehat{\sigma }_{Xkj}$ for an omics biomarker $k = 1,2,3$ and genetic variant $j$

$$\begin{align*} & X_{k} \sim \beta_{0Xkj} + \beta_{Xkj} Z_{j}. \end{align*}$$

For the association between outcome $Y$ and genetic variant $Z_j$, when we have binary outcome $Y_B$, we fit the logistic regression model to get effect size estimate $\widehat{\beta }_{Yj}$ and standard error estimate $\widehat{\sigma }_{Yj}$

$$\begin{align*} & \mathrm{logit}(Y_B) \sim \beta_{0Yj} + \beta_{Yj} Z_{j}, \end{align*}$$

and when we have continuous outcome $Y_C$, we fit the regression model to get effect size estimate $\widehat{\beta }_{Yj}$ and standard error estimate $\widehat{\sigma }_{Yj}$

$$\begin{align*} & Y_C \sim \beta_{0Yj} + \beta_{Yj} Z_{j}. \end{align*}$$

Below is a list of simulation settings.

(i) Pleiotropy: horizontal or vertical (Figure 5);
(ii) Number of SNPs used as IVs: 5 or 20;
(iii) Overlap between samples involved in the GWAS studies and the QTL studies: 0 (two-sample), 0.5 (half of the samples overlap) or 1 (one-sample);
(iv) Use same samples or independent samples to calculate QTLs belonging to multiple omics biomarkers under a two-sample setting;
(v) Outcome: continuous ($Y_C$) or binary ($Y_B$). When the outcome is binary, we also need to decide whether we will only use control samples to estimate the QTLs;
(vi) LD between different SNPs $r^2$: 0, 0.01 or 0.2;
(vii) Strength of IV $\alpha $, which is proportional to the association between IVs and exposures: 0.5, 1 or 2;
(viii) Effect size of the pleiotropic association between exposure and outcome $\beta _X$: 0 or 0.1.

We set $\beta _X = 0.1$ (positive casual effect) to evaluate the power and set $\beta _X = 0$ (null casual effect) to evaluate the type I error. We set the IV strength $\alpha $ to be either 0.5, 1 or 2.

### Comparison of type I error across methods

**Figure 3:**
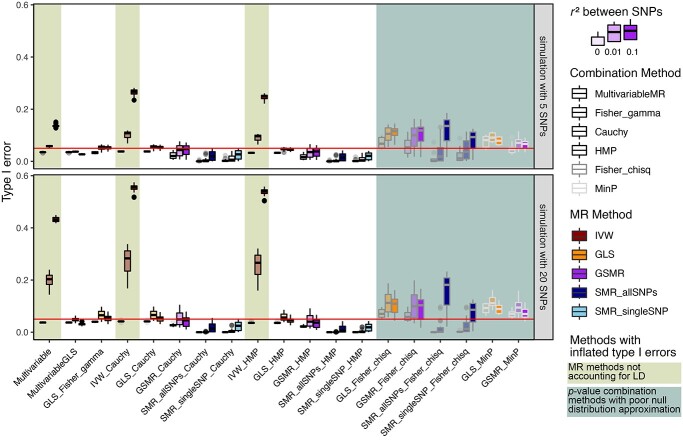
A comparison of type I error rates across combinations of MR methods and combination methods. A method is disqualified if its type I error rate exceeds the nominal significance level of 0.05 (red horizontal line). This can be due to a failure in correcting for LD between SNPs (labeled in light green) or poor approximation of null distribution when the underlying $P$-values are correlated, including Fisher combination function using chi-squared distribution (Fisher_chisq) and minimum $P$-value (MinP) (labeled in light blue). Each observation in the boxplot represents the type I error rate of a simulation setting with 10 000 replications. Simulation settings are included if either (1) outcome is binary and only control samples are used when estimating QTL or (2) when there is no overlap between samples in the QTL and the GWAS datasets.

**Figure 4:**
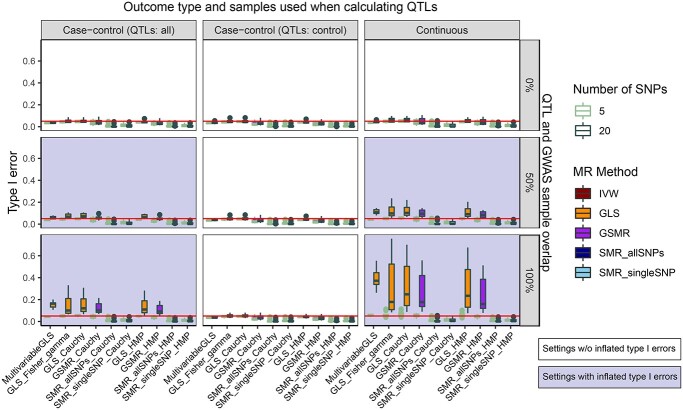
Type I error rate of combinations of MR methods and combination methods when the QTL and the GWAS datasets are simulated with various degrees of overlap between samples (rows of the grid) and outcome types being continuous or binary (columns of the grid). Simulation settings that have inflated type I errors are labeled in light purple. Each observation in the boxplot represents the type I error rate under a simulation scenario with 10 000 replications. MR methods that are not able to account for correlation between SNPs and/or correlation between $P$-values of MR analyses conducted in different exposures are excluded. The red horizontal line depicts the nominal significance level of 0.05.

In the subsequent simulations, we eliminate the methods that fail to control type I error (methods with a shaded background in Figures 2A and 3) and identify the ones whose powers stand out from the pack after another round of competition in Figure 4.

First, we verify whether GLSs help us adjust for LD between SNPs used as IVs after pre-emptively striking out some scenarios where the excessive participants overlap between GWAS and QTL studies could bring bias.

Figure 3 illustrates that when the LD is not accounted for in the MR analysis, the type I error tends to increase sharply especially when there is a larger number of SNPs (20 vs. 5), even the true LD between SNPs is only 0.01. Given the difficulty of accurately estimating the LD matrix, the most prudent course of action is to select SNPs known to be uncorrelated to use as IVs, which is not practical since the association between an SNP and an exposure is often local and we may fail to identify a considerable number of independent SNPs as valid IVs. The comparison of methods stresses that a valid method needs to account for LD between SNPs using GLSs under regression-based models.

Then, we verify whether there is an inflated type I error rate associated with the use of several $P$-value combination methods. The $P$-value combination methods need to be valid in the presence of correlation between $P$-values. Observed from left to right of the $x$-axis labels of Figure 3, Cauchy, HMP and Fisher combination method with a gamma distribution approximation (Fisher_gamma) can control type I error. This is expected because both the HMP and the Cauchy combination method work regardless of the correlation between $P$-values. Meanwhile, Fisher combination method with a chi-squared distribution approximation (Fisher_chisq), which does not adjust for correlation between $P$-values, and the minimum *P*-value method (MinP) both fail to control type I error.

### Resolution to sample overlap between GWAS and QTL studies

We discuss what level of sample overlap between GWAS and QTL studies is appropriate to be incorporated in an MR analysis. It has been reported in the literature that the causal effect size estimate in MR will be biased towards the OLS estimate, which is the direction of there being no effect when the samples in the GWAS and the samples in QTL studies have no overlap since they are independent (‘two-sample’). When there is considerable overlap between samples involved in the GWAS and QTL studies (‘one-sample’), however, the MR causal effect size estimate will be biased towards the direction of there being an effect, which is usually nonzero under the presence of the confounders [28].

Our findings mostly align with what is reported in the literature with some subtleties. In Figure 4, when the overlap is 1, which means the samples underlying the GWAS and QTL studies are the same, there is an inflated type I error for the methods that rely on a model-based approach to integrate the information from multiple SNPs, and the symptom is more prominent when the number of SNPs is 20 instead of 5. The methods based on combining $P$-values arising from one-SNP-based SMR methods can control type I errors surprisingly well considering that SMR requires two samples. Other than the obvious corollary that we need to pick a GWAS dataset without sample overlap with QTL datasets, we can also bring type I error under control when the outcome is binary and only control samples are considered when estimating the QTLs.

### Comparison of power across methods

**Figure 5:**
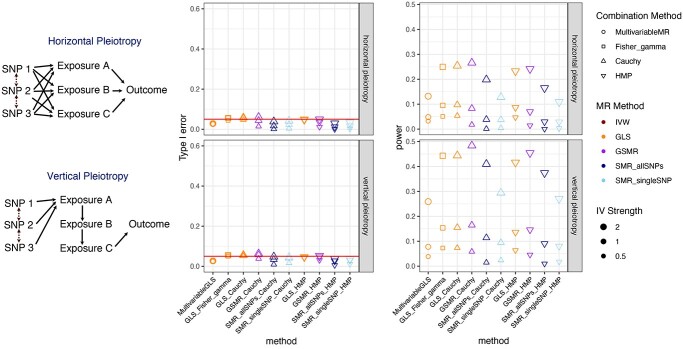
Type I error rate (middle column) and power (right column) of selected combinations of MR methods and combination methods whose empirical type I error rates do not exceed the nominal significance level. The first row of panels represents the results under horizontal pleiotropy and the second row of panels represents the results under vertical pleiotropy. The number of SNPs in the simulation is 5 (only 3 SNPs are depicted in the diagram due to space constraints) and the $r^2$ between the SNPs is 0.2. In the simulation, there is no overlap between samples used in QTL and GWAS datasets, and both case and control samples are used when estimating QTLs. Each point represents the positive rate under a simulation scenario with 10 000 replications. The red horizontal line depicts the nominal significance level of 0.05.

After we narrow down the candidate methods to those will not bring inflated type I errors, we pit them against each other and figure out whose power is the highest. In Figure 5, we find that GLS_Fisher_gamma and GLS_Cauchy are the most powerful ones throughout different scenarios, and GSMR_Cauchy is about as powerful when the IVs are strong but is less powerful with weak IVs. We also confirm that the methods being examined will not produce inflated type I error rates.

We verify if our combination method works under different genetic architecture of traits through additional simulations. Similar type I error rates and powers can be observed when

(i) The number of SNPs changes from 5 to 20 (Supporting Figure 1);
(ii) The causal effects of SNPs on omics biomarkers follow i.i.d. beta distributions instead of being constant (Supporting Figures 3, 4, and Web Appendix B);
(iii) The phenotype and the omics biomarkers are simulated using real genotypes from the 1000 Genomes Project (Supporting Figure 6 and Web Appendix C).

These results provide additional supporting evidence for the utility of our proposed framework.

## Prioritization of genes linked to Alzheimer’s disease using expression, proteomics and metabolomics biomarkers

### Study samples and real datasets

We analyze multi-omics data related to Alzheimer’s disease to assess the performance of different methods on identifying important genes where the omics biomarkers have pleiotropic associations with outcome. Data used in our analysis were obtained from the Alzheimer’s Disease Neuroimaging Initiative (ADNI) database (adni.loni.usc.edu). ADNI was launched in 2003 as a public–private partnership to test whether serial MRI, PET and biological markers can be combined with clinical and neuropsychological assessments to accurately measure the progression of mild cognitive impairment (MCI) and early AD. For up-to-date information, see www.adni-info.org. We use genotype, methylation and metabolomics data derived from members of the ADNI cohort. See Web Appendix A in Supporting Information for how the QTLs of omics data are calculated [29].

### Downloading GWAS summary data

The GWAS summary data are obtained from a meta-analysis study [30] of European ancestry individuals. This study integrates the GWAS summary statistics from Kunkle *et al.* [31] (22 000 cases and 42 000 controls) with the GWAX (GWAS by proxy) summary statistics in UKB (53 000 proxy cases and 378 000 controls). Individuals were denoted as ‘cases’ if either of their or any siblings had AD. The data were downloaded from http://ftp.ebi.ac.uk/pub/databases/gwas/summary_statistics/GCST90012001-GCST90013000/GCST90012877/.

### Steps of real data analysis

We further analyze the real datasets described earlier to assess the performance of the proposed methods.

(i) Prepare gene-level metQTL summary data by mapping the metabolites to genes using KEGG metabolic pathways. If several metabolites in SNP-metabolite associations are mapped to one gene, the metQTL to be retained is the one with the smallest $P$-value.
(ii) Prepare gene-level pQTL summary data by mapping the UNIPROT AC/ID to Gene name on https://www.uniprot.org/uploadlists/.
(iii) Merging downloaded metQTL and pQTL into the eQTL meta-analysis summary statistics by aligning SNP IDs and gene symbols.
(iv) Select a list of 584 genes with some evidence of association to Alzheimer’s disease from Agora genes at https://agora.adknowledgeportal.org/genes.
(v) Within the Agora genes, select 41 genes where all three omics biomarkers (expression, proteomics and metabolomics) are present.
(vi) Select 27 genes that at least one of eQTL, pQTL and metQTL is significant ($P$-value $< \frac{0.05}{41}$).
(vii) For each gene, select cis-SNPs (distance between gene and SNP less than 100 kb) and perform LD clumping in each omics biomarker (select SNP with the smallest QTL when possible, $r^2 < 0.2$, distance > 100 kb).
(viii) Perform MR analysis for each omics biomarker using the exposure datasets and the outcome datasets.
(ix) Integrate $P$-values using combination tests.

### Results

In this real example of a multi-omics MR analysis, we first decide if we should proceed with a one-sample or a two-sample framework. The former corresponds to using QTL datasets whose participants overlap those in the GWAS dataset, while the latter requires QTL datasets unrelated to the GWAS dataset. The main argument for the two-sample MR framework is that the sample size of the GWAS dataset is several magnitudes larger, enabling more discoveries. Another benefit is that we are not plagued by the over-inflated type I error rate that would arise in a one-sample framework unless the QTL summary data are derived solely from control subjects. Therefore, we aim to use an external GWAS dataset (meta-analysis of AD by proxy [30]) to perform a two-sample MR.

We start from a list of 584 ‘Nominated Targets’ from the Agora website [32], which include genes that are potential targets of Alzheimer’s disease identified through genomic, proteomic and/or metabolomic data analysis. After selecting the genes that are covered by the expression, proteomic and metabolomic assays, we have 41 genes. Next, we stipulate that any genes left would need to have at least one of eQTL, pQTL and metQTL being significant (Bonferroni-corrected $P$-value $< 0.05 / 41$), so the associated SNPs can be selected as IVs, and 27 genes survive this threshold. Then, we perform a targeted analysis using these prominent gene targets.

**Figure 6:**
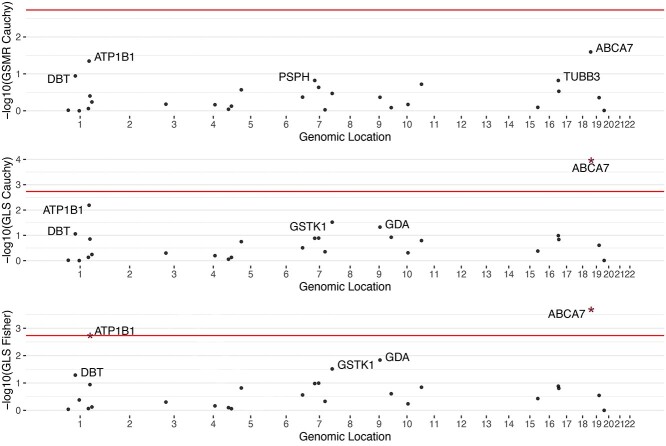
The $P$-values of 27 Alzheimer’s disease-related genes in the integrated analysis of GWAS, eQTL, pQTL and metQTL. Features whose test for pleiotropy association between omics biomarker (exposure) and outcome passed a genome-wide significance level (red horizontal line) after Bonferroni adjustment for 27 genes are marked using a violet asterisk *. (Top panel) $P$-value in tests using GLSs for each omics biomarker. (Middle anel) Combined GLS $P$-values each representing one of the three omics biomarkers using Cauchy combination test. (Bottom panel) Combined GLS $P$-values each representing one of the three omics biomarkers using Fisher combination function and gamma distribution approximation accounting for correlation between the underlying $P$-values.

Fisher combination test using a gamma distribution approximation identifies two significant findings marked using an asterisk ${}^\ast $ (Figure 6). The panels illustrate three different methods combining MR results on each gene with mapped eQTL, pQTL and metQTL. The horizontal red line represents the cutoff of a significance level of 0.05 after adjusting for multiple comparisons using the Bonferroni method. Focusing on the genes that pass the cutoffs, the gene *ABCA7* is the most significant finding when using either the Cauchy combination test or Fisher combination test. There are no other genome-wide significant findings in the Cauchy combination test, while the Fisher combination test (using a Gamma distribution approximation) picks another significant gene in *ATP1B1*.

Both *ABCA7* and *ATP1B1* are candidate biomarkers of the Alzheimer’s disease. *ABCA7* is a protein-encoding gene that creates ATP-binding cassette sub-family A member 7. This protein is one of the ATP-binding cassette (ABC) transporters, which generally transports molecules across cellular membranes [33]. To date, two large genome-wide association studies (GWAS) [34, 35] have implicated *ABCA7* as a susceptibility locus for late-onset Alzheimer’s Disease. Specifically, *ABCA7* has been associated with memory decline [36], incident MCI [36] and scortical/hippocampal atrophy [37]. These findings were also biologically verified using knock-out mice: deletion of *ABCA7* resulted in the increased production of A-beta proteins indicative of AD [38, 39]. The *ATP1B1* gene encodes the eponymous protein *ATP1B1* (sodium/potassium-transporting ATPase subunit beta-1) [40]. This protein is part of an enzyme that catalyzes the hydrolysis of ATP which is coupled with the exchange of Na$^+$ and K$^+$ ions across the plasma membrane. The beta subunit this protein contributes to regulates the quantity of sodium pumps transported to the plasma membrane and is therefore essential to neuron function and health [41].

Other combination tests and multivariable MR fail to report any significant findings after a multiple adjustment. Using the GSMR approach with the Cauchy combination test, the smallest $P$-value among the 27 tests turns out to be 0.0254, which is not genome-wide significant after Bonferroni adjustment for 27 tests. This agrees with the simulation result in Figure 5: when the IVs are weaker, the GSMR approach is less powerful compared with GLS. The GSMR being less powerful is partly due to mostly only two or three IVs left after the LD clumping, which fell short of the cutoff in the GSMR implementation recommending at least 10 strong IVs. Likewise, the application of multivariable MR requires an even more stringent number of IVs. The test can proceed only if there are least four IVs left after the LD clumping. As a result, out of 27 genes we tested, only in two genes multivariable MR produces non-NA $P$-values, both of which are not significant.

## Discussion

MR methods can identify pleiotropic association using GWAS and QTL summary data. Pleiotropy happens when an SNP has multiple effects, and it is widespread in MR investigations considering that an SNP can affect more than one omics biomarker. When QTL data from multiple omics biomarkers are available, the MR analyses that focused on one gene can be integrated using $P$-value combination methods. Some $P$-value combination methods can account for the correlation between the MR test statistics. They include the Cauchy combination test, harmonized mean $P$-value (HMP) and Fisher combination function with gamma distribution approximation. The simulations and real data analysis favor Fisher combination function with gamma distribution approximation, especially when the IVs are weak.

We explore how multi-omics data can be integrated into the MR framework. Pleiotropy, which happens when an SNP has multiple effects, is very common in multi-omics studies. Previously, researchers distinguished vertical pleiotropy from horizontal pleiotropy (Figure 5). Vertical pleiotropy can be investigated using multi-step MR by picking exposure and outcome as omics-data types where we want to confirm a causal relationship [42]. On the other hand, multivariable MR suits horizontal pleiotropy better where the independent effects of multiple exposures can be estimated in one model. Our novel approach focuses on combining multiple $P$-values after performing MR in each -omics biomarker. Such prioritization of genes in a genome-wide analysis achieves an increase in power at the expense of deeper interrogations of relationship between biomarkers.

In our MR experiment investigating GWAS and QTLs, we consider cis-QTLs which are associations between variants and exposure at most one megabase pairs apart. For an exposure, there might not be multiple SNPs with significant cis-QTLs after LD clumping of the candidate SNPs. Considering that a majority of SNPs affect the multi-omic exposures locally, we usually do not have multiple independent SNPs as IVs due to LD, restricting our capability of further interpreting MR results. Therefore, it is usually not practical to tell apart causality from pleiotropy and linkage [7].

Apart from one-sample and two-sample MR, there is another approach known as three-sample MR [43], whereby we can use selection datasets with no overlap in the individuals compared with the exposure datasets. If we use the same datasets both to select the SNPs and to provide statistics to perform MR analysis as shown in the real data analysis example, we may suffer from the ‘winner’s curse’ and generally have a causal effect size estimate biased towards zero. A future direction to our proposed strategy is to adopt a three-sample approach by finding other eQTL, pQTL and metQTL datasets for the MR analysis.

We note that the mediation analysis model attempts to evaluate the joint effect of genotype on outcome [44, 45]. MR is also compared with fine mapping and colocalization [46], both methods working on a single gene region, where fine mapping strives to find causal genetic variants for a trait and colocalization aims to identify variants that are associated with both traits. The concept of MR is unique in that it quantifies the causal relationship from exposure to outcome (if the assumptions are satisfied and the effect is not due to pleiotropy or linkage) and the exposures may serve as targets for intervention [47].

In conclusion, we developed a framework for summary-data-based MR analysis where multiple omics biomarkers can be viewed as multiple exposures, with an emphasis on the combination tests and special handling due to correlated $P$-values from single-exposure MR tests. While our proposed framework does not assume the dependence structure between the omics biomarkers, we anticipate that it can be complemented by specifically designed tests that can untangle the effect of each omics biomarker in future research.

**Key Points**

- We propose combination tests that aggregate $P$-values related to a gene after Mendelian randomization (MR) analyses probing the causal effect (or pleiotropic effect) of omics biomarkers on the outcome.
- Both in simulations and a real example, the combination tests are more powerful in gene prioritization than the multivariable MR framework.
- We recommend using Fisher combination test with gamma distribution approximation. Its power is the highest among the compared methods especially when only weak IVs can be selected from the variants.

## Data availability statement

The source code and supporting data are available through GitHub (https://github.com/lshen/adomics). Processed eQTL data used in the analysis are from the ROS/MAP, MSBB and MayoRNAseq studies and are available after application through the AMP-AD knowledge portal (https://adknowledgeportal.synapse.org) [48]. Processed pQTL data are from the ROS/MAP project [49]. Methylation data, metabolomics data and genotypes used to generate meQTL and metQTL in this analysis are from the ADNI project and are available after application through the Alzheimer’s Disease Neuroimaging Initiative website (http://adni.loni.usc.edu/).

## Authors’ contributions statement

Q.L. and L.S. initiated the study. Q.L., L.S. and C.J. designed the study. B.L. and C.J. processed real data. C.J. implemented the methods, benchmarked them on simulations and real data and generated visualizations. C.J., B.L., L.S., and Q.L. wrote, revised and reviewed the manuscript.

## Author Biographies

**Chong Jin** is a postdoctoral researcher at the University of Pennsylvania. His research focuses on developing statistical methods for the integrative analysis of multi-omics data.

**Brian Lee** is a graduate student at the University of Pennsylvania working under Prof. Li Shen. His research focuses on the application of statistical techniques to analyze multi-omic and biomedical data.

**Li Shen** is a Professor of Informatics at the University of Pennsylvania. His research focuses on developing and applying informatics and computing methods for analyzing genetics, omics, imaging, cognition, and other biomedical and health data.

**Qi Long** is a Professor of Biostatistics at the University of Pennsylvania. His research focuses on developing robust statistical and machine learning methods for analysis of complex health data (such as -omics and electronic health records data), missing data, causal inference, data privacy and fairness.
